# Supplementary material for: MEK reduces cancer-specific PpIX accumulation through the RSK-ABCB1 and HIF-1α-FECH axes
Source: Sci Rep. 2020 Dec 17;10:22124. doi: 10.1038/s41598-020-79144-x (PMC7747616; doi:10.1038/s41598-020-79144-x)
Supplement: Supplementary file 1 — Supplementary Information 1. [file 41598_2020_79144_MOESM1_ESM.pdf]

Supplementary Information

MEK reduces cancer-specific PpIX accumulation through the RSK-ABCB1 and HIF-1α-FECH axes

Vipin Shankar Chelakkot[1], Kaiwen Liu[1], Ema Yoshioka[1], Shaykat Saha[1], Danyang Xu[1], Maria Licursi[1], Ann Dorward[1,] and Kensuke Hirasawa[1]*

[1]Division of BioMedical Sciences, Faculty of Medicine, Memorial University of Newfoundland, St. John's, NL, Canada, A1B 3V6

Running title: RSK and HIF-1α activation reduces PpIX accumulation

***To whom correspondence should be addressed: Kensuke Hirasawa, PhD**; Division of BioMedical Sciences, Faculty of Medicine, Memorial University of Newfoundland, 300 Prince Philip Drive, St. John's, NL, Canada, A1B 3V6, Phone: +1 (709) 864-6058, Email: kensuke@mun.ca

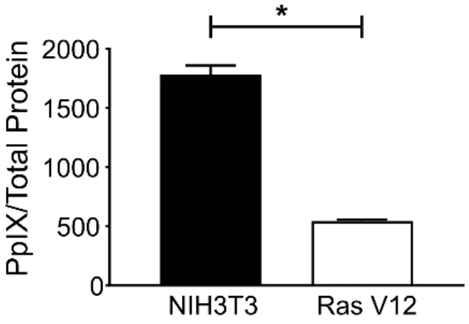

Supplementary Figure S1. NIH3T3 and Ras V12 cells were treated with 5 mM 5-ALA for 4 h, and the PpIX fluorescence in the cell lysates was measured using a fluorescence plate reader (BioTek Instruments Inc. VT) with a 405 nm excitation/630 nm emission filter. The total protein in the lysate was measured using a BCA protein assay kit (Thermo Scientific). PpIX fluorescence was then normalised to the total protein. The plot shows the mean ± SD normalised PpIX fluorescence from 3 independent experiments. *p<0.01 by Student's *t*-test.

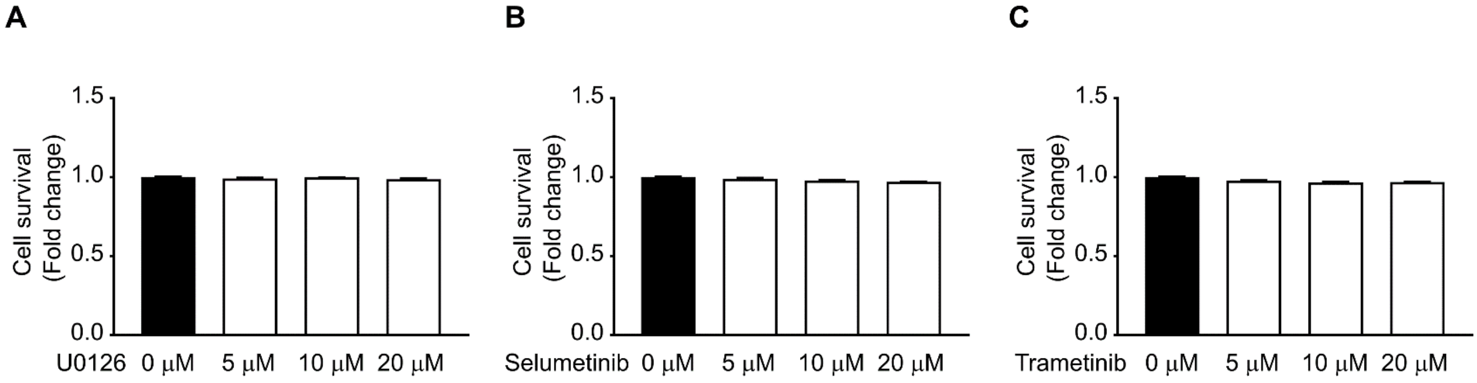

Supplementary Figure S2. RasV12 cells were treated with different concentrations of (A) U0126, (B) Selumetinib, or (C) Trametinib for 24 h. The cell viability was measured using the Colorimetric Cell Viability Kit I (WST-8) (PromoCell GmbH, Germany), following the manufacturer's instructions. The plots show the mean ± SD fold change in cell survival.

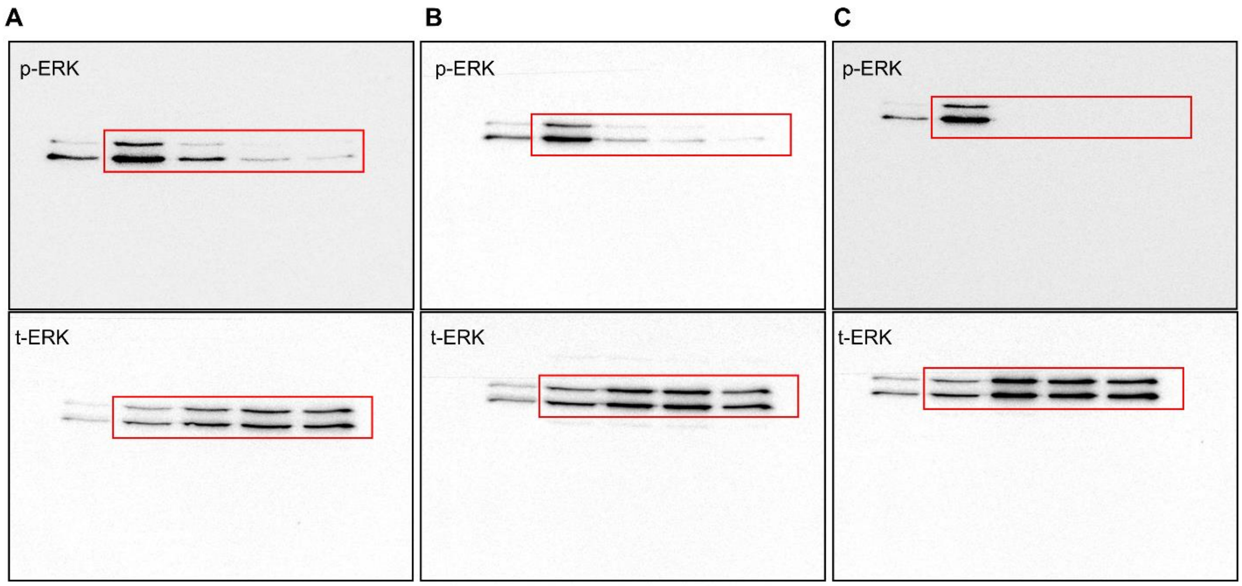

Supplementary Fig S3. Uncropped immunoblot for Fig 2A (A), 2B (B), and 2C (C).

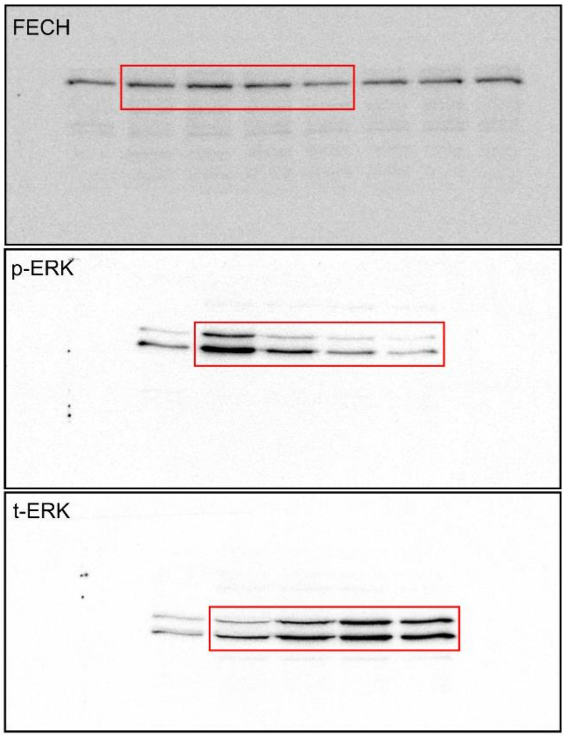

Supplementary Fig S4. Uncropped immunoblot for Fig 4D.

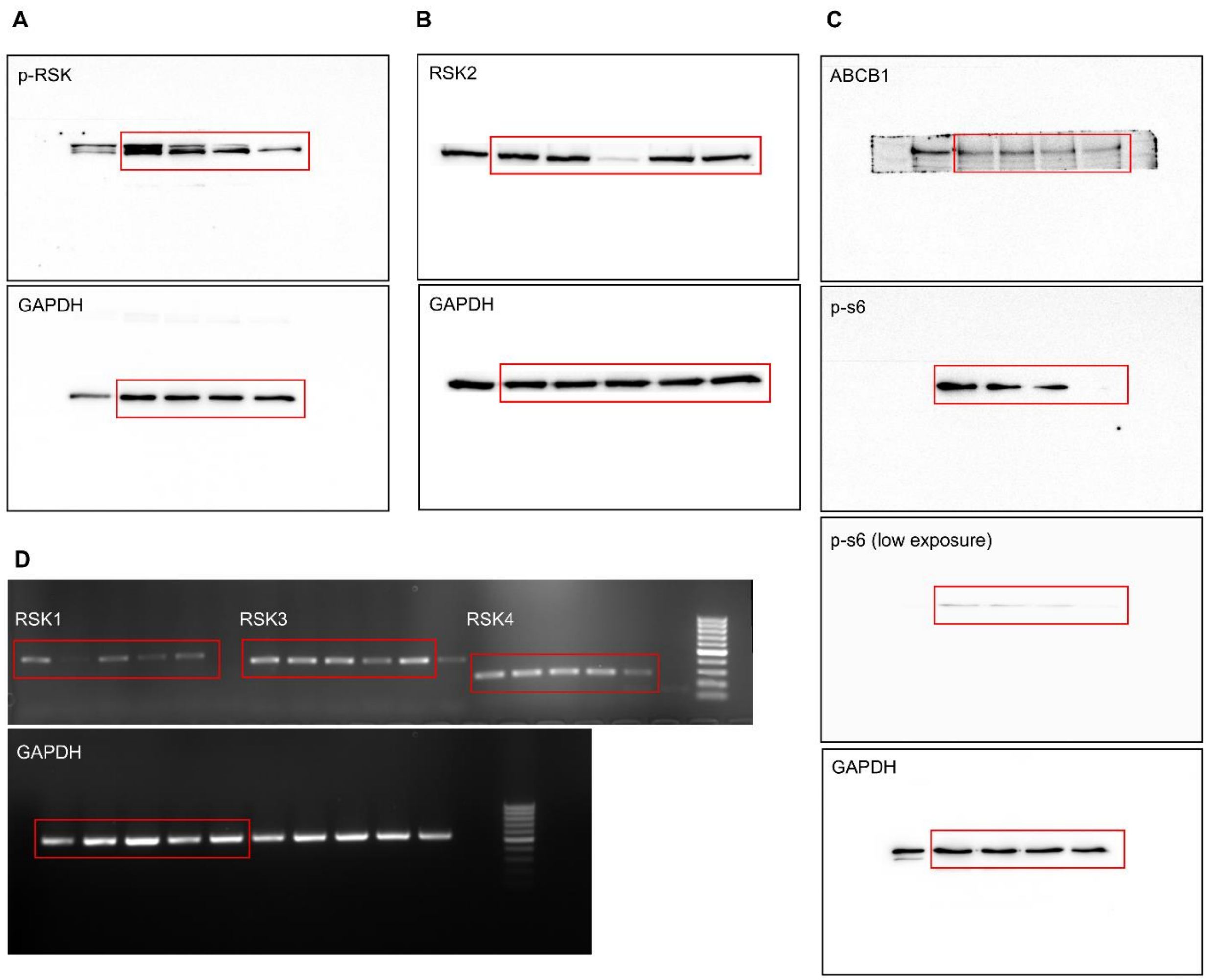

Supplementary Fig S5. Uncropped immunoblots for Fig 5A (A), 5B (B), and 5E (C), and uncropped gel image for Fig 5B (D). The RSK2 and ABCB1 blots were cut before imaging to avoid interference from bright non-specific bands.

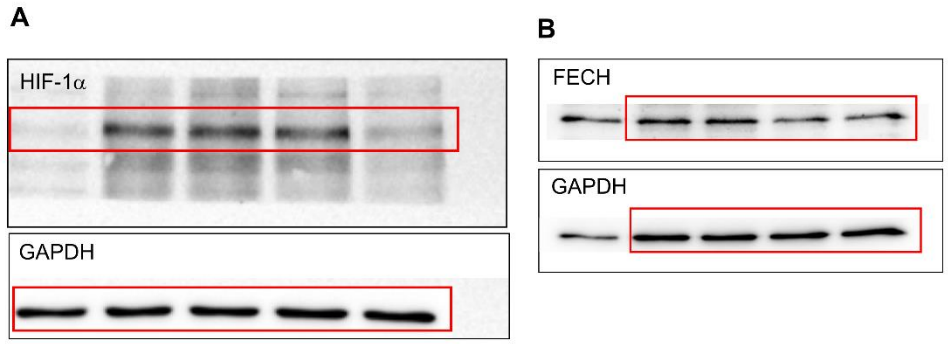

Supplementary Fig S6. Uncropped immunoblot for Fig 6A (A) and 6B (B). The FECH blot was cut before imaging to avoid interference from bright non-specific bands.
